# Alcoholism Identification Based on an AlexNet Transfer Learning Model

**DOI:** 10.3389/fpsyt.2019.00205

**Published:** 2019-04-11

**Authors:** Shui-Hua Wang, Shipeng Xie, Xianqing Chen, David S. Guttery, Chaosheng Tang, Junding Sun, Yu-Dong Zhang

**Affiliations:** ^1^School of Computer Science and Technology, Henan Polytechnic University, Jiaozuo, China; ^2^School of Architecture Building and Civil Engineering, Loughborough University, Loughborough, United Kingdom; ^3^Department of Informatics, University of Leicester, Leicester, United Kingdom; ^4^College of Telecommunications and Information Engineering, Nanjing University of Posts and Telecommunications, Nanjing, China; ^5^Department of Electrical Engineering, College of Engineering, Zhejiang Normal University, Jinhua, China; ^6^Guangxi Key Laboratory of Manufacturing System and Advanced Manufacturing Technology, Guilin, China

**Keywords:** alcoholism, transfer learning, AlexNet, data augmentation, convolutional neural network, dropout, local response normalization, magnetic resonance imaging

## Abstract

**Aim:** This paper proposes a novel alcoholism identification approach that can assist radiologists in patient diagnosis.

**Method:** AlexNet was used as the basic transfer learning model. The global learning rate was small, at 10^−4^, and the iteration epoch number was at 10. The learning rate factor of replaced layers was 10 times larger than that of the transferred layers. We tested five different replacement configurations of transfer learning.

**Results:** The experiment shows that the best performance was achieved by replacing the final fully connected layer. Our method yielded a sensitivity of 97.44%± 1.15%, a specificity of 97.41 ± 1.51%, a precision of 97.34 ± 1.49%, an accuracy of 97.42 ± 0.95%, and an F1 score of 97.37 ± 0.97% on the test set.

**Conclusion:** This method can assist radiologists in their routine alcoholism screening of brain magnetic resonance images.

## Introduction

Alcoholism (1) was previously composed of two types: alcohol abuse and alcohol dependence. According to current terminology, alcoholism differs from “harmful drinking” (2), which is an occasional pattern of drinking that contributes to increasing levels of alcohol-related ill-health. Today, it is defined depending on more than one of the following conditions: alcohol is strongly desired, usage results in social problems, drinking large amounts over a long time period, difficulty in reducing alcohol consumption, and usage resulting in non-fulfillment of everyday responsibilities.

Alcoholism affects all parts of the body, but it particularly affects the brain. The size of gray matter and white matter of alcoholism subjects are less than age-matched controls (3), and this shrinkage can be observed using magnetic resonance imaging (MRI). However, neuroradiological diagnosis using MR images is a laborious process, and it is difficult to detect minor alterations in the brain of alcoholic patient. Therefore, development of a computer vision-based automatic smart alcoholism identification program is highly desirable to assist doctors in making a diagnosis.

Within the last decade, studies have developed several promising alcoholism detection methods. Hou (4) put forward a novel algorithm called predator-prey adaptive-inertia chaotic particle swarm optimization (PAC-PSO), and applied it to identify alcoholism in MR brain images. Lima (5) proposed to use Haar wavelet transform (HWT) to extract features from brain images, and the authors used HWT to detect alcoholic patients. Macdonald (6) developed a logistic regression (LR) system. Qian (7) employed the cat swarm optimization (CSO) and obtained excellent results in the diagnosis of alcoholism. Han (8) used wavelet Renyi entropy (WRE) to generate a new biomarker; whereas Chen (9) used a support vector machine, which was trained using a genetic algorithm (SVM-GA) approach. Jenitta and Ravindran (10) proposed a local mesh vector co-occurrence pattern (LMCoP) feature for assisting diagnosis.

Recently, deep learning has attracted attention in many computer vision fields, e.g., synthesizing visual speech (11), liver cancer detection (12), brain abnormality detection (13), etc. As a result, studies are now focused on using deep learning techniques for alcoholism detection. Compared to manual feature extraction methods (14–18), deep learning can “learn” the features of alcoholism. For example, Lv (19) established a deep convolutional neural network (CNN) containing seven layers. Their experiments found that their model obtained promising results, and the stochastic pooling provided better performance than max pooling and average pooling. Moreover, Sangaiah (20) developed a ten-layer deep artificial neural network (i.e., three fully-connected layers and seven conv layers), which integrated advanced techniques, such as dropout and batch normalization, into their neural network.

Transfer learning (TL) is a new pattern recognition problem-solver (21–23). TL attempts to transfer knowledge learned using one or more source tasks (e.g., ImageNet dataset) and uses it to improve learning in a related target task (24). In perspective of realistic implementation, the advantages of TL compared to plain deep learning are: (i) TL uses a pretrained model as a starting point; (ii) fine-tuning a pretrained model is usually easier and faster than training a randomly-initialized deep neural network.

The contribution of this paper is that we may be the first to apply transfer learning in this field of alcoholism identification. We used AlexNet as the basic transfer learning model and tested different transfer configurations. Further, the experiments showed that the performance (sensitivity, specificity, precision, accuracy, and F1 score) of our model is >97%, which is superior to state-of-the-art approaches. We also validated the effectiveness of using data augmentation which further improves the performance of our model.

## Data Preprocessing

### Datasets

This study was approved by the ethical committee of Henan Polytechnic University. Three hundred seventy-nine slices were obtained in which there are 188 alcoholic brain images and 191 non-alcoholic brain images. We divided the dataset into three parts: a training set containing 80 alcoholic brain images and 80 non-alcoholic brain images; A validation set containing 30 alcoholic brain images and 30 non-alcoholic brain images; a test set containing 78 alcoholic brain images and 81 non-alcoholic brain images. The division is shown in Table 1.

**Table 1:** Dataset division into training, validation, and test sets.

	**Alcoholic**	**Non-alcoholic**	**Total**
Training	80	80	160
Validation	30	30	60
Test	78	81	159
Total	188	191	379

### Data Augmentation

To improve the performance of deep learning, data augmentation (DA) (25) was introduced. This was done because our deep neural network model has many parameters, so we needed to show that our model contains a proportional amount of sample images to achieve optimal performance. For each original image, we generated a horizontally flipped image. Then, for both original and horizontal-flipped images, we applied the following five DA techniques: (i) noise injection, (ii) scaling, (iii) random translation, (iv) image rotation, and (v) gamma correction. Each of those methods produced 30 new images.

Gaussian noise with zero-mean and variance of 0.01 was applied to every image. Scaling was used with a scaling factor of 0.7–1.3, with an increase of 0.02. Random translation was utilized with a random shift within [−40 40] pixels. Image rotation with rotation angle varies from −30^o^ to 30^o^ and a step of 2^o^ was employed. Gamma correction with gamma value varies from 0.4 to 1.6 with a step of 0.04 was utilized.

The DA result is shown in Table 2. Each image generated (1+30^*^5)^*^2 = 302 images including itself. After DA, the training set had 24,160 alcoholism brain images and 24,160 healthy brain images. Altogether, the new training set consisted of a balanced 160^*^320 = 48,320 samples.

**Table 2:** Data augmentation.

	**Alcoholic**	**Non-alcoholic**	**Total**
Original Image	80	80	160
DA_I: Noise Injection	2,400	2,400	4,800
DA_II: Scaling	2,400	2,400	4,800
DA_III: Random Translation	2,400	2,400	4,800
DA_IV: Image Rotation	2,400	2,400	4,800
DA_V: Gamma Correction	2,400	2,400	4,800
Horizontal-flipped Image	80	80	160
DA_I: Noise Injection	2,400	2,400	4,800
DA_II: Scaling	2,400	2,400	4,800
DA_III: Random Translation	2,400	2,400	4,800
DA_IV: Image Rotation	2,400	2,400	4,800
DA_V: Gamma Correction	2,400	2,400	4,800
New Training Data	24,160	24,160	48,320

## Methodology

### Fundamentals of Transfer Learning

**Figure 1:**
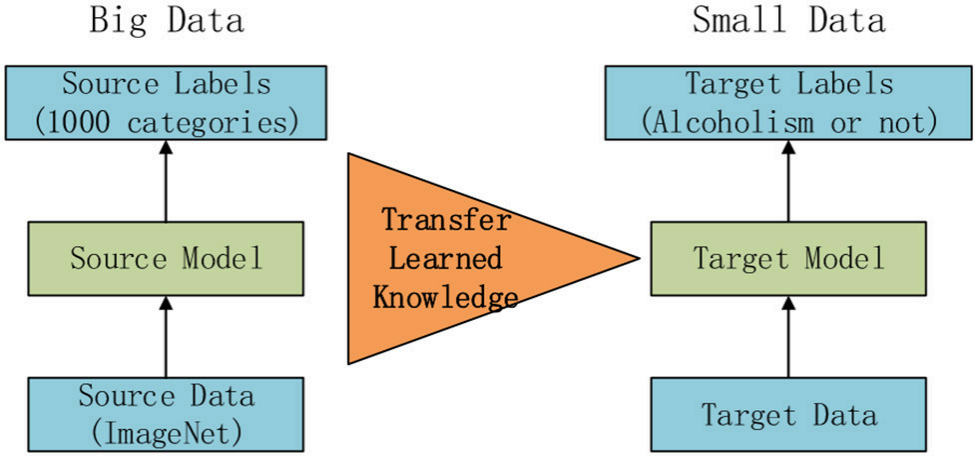
Idea of transfer learning.

The core knowledge of transfer learning (TL) is shown in Figure 1. The core is to use a relatively complex and successful pre-trained model, trained from a large data source, e.g., ImageNet, which is the large visual database developed for visual object recognition research (26). It contains more than 14,000,000 hand-annotated images and at least one million images are provided with bounding boxes. ImageNet contains more than 20,000 categories (27). Usually, pretrained models are trained on a subset of ImageNet with 1,000 categories. Then we “transferred” the learnt knowledge to the relatively simplified tasks (e.g., classifying alcoholism and non-alcoholism in this study) with a small amount of private data.

Two attributes are important to help the transfer (28): (i) The success of the pretrained model can promote the exclusion of user intervention with the boring hyper-parameter tuning of new tasks; (ii) The early layers in pretrained models can be determined as feature extractors that help to extract low-level features, such as edges, tints, shades, and textures.

Traditional TL only retrains the new layers (29). In this study, we initially used the pretrained model, and then re-trained the whole structure of the neural network. Importantly, the global learning rate is fixed, and the transferred layers will have a low factor, while newly-added layers will have a high factor.

### AlexNet

**Figure 2:**
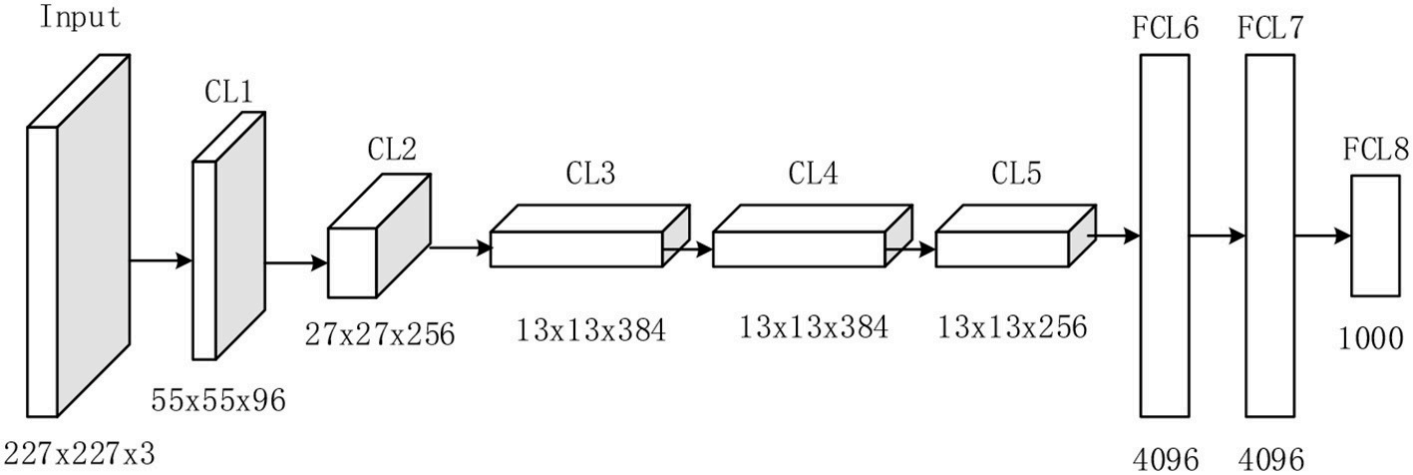
Structure of AlexNet (5 CLs and 3 FCLs).

AlexNet competed in the ImageNet challenge (30) in 2012, achieved a top-5 error of only 15.3%, more than 10.8% better than the result of the runner-up that used the shallow neural network. Original AlexNet was performed on two graphical processing units (GPUs). Nowadays, researchers tend to use only one GPU to implement AlexNet. Figure 2 illustrates the structure of AlexNet. This study only counts layers associated with learnable weights. Hence, AlexNet contains five conv layers (CL) and three fully-connected layers (FCL), totaling eight layers.

The details of learnable weights and biases of AlexNet are shown in Table 3. The total weights and biases of AlexNet are 60,954,656 + 10,568 = 60,965,224. In Matlab, the variable is stored in single-float type, taking four bytes for each variable. Hence, in total we needed to allocate 233 MB.

**Table 3:** Learnable layers in AlexNet.

**Name**	**Weights**	**Biases**
CL1	11*11*3*96 = 34,848	1*1*96 = 96
CL2	5*5*48*256 = 307,200	1*1*256 = 256
CL3	3*3*256*384 = 884,736	1*1*384 = 384
CL4	3*3*192*384 = 663,552	1*1*384 = 384
CL5	3*3*192*256 = 442,368	1*1*256 = 256
FCL6	4096*9216 = 37,748,736	4096*1 = 4,096
FCL7	4096*4096 = 16,777,216	4096*1 = 4,096
FCL8	1000*4096 = 4,096,000	1000*1 = 1,000
CL Subtotal	2,332,704	1,376
FCL Subtotal	58,621,952	9,192
Total	60,954,656	10,568

### Common Layers in AlexNet

**Figure 3:**
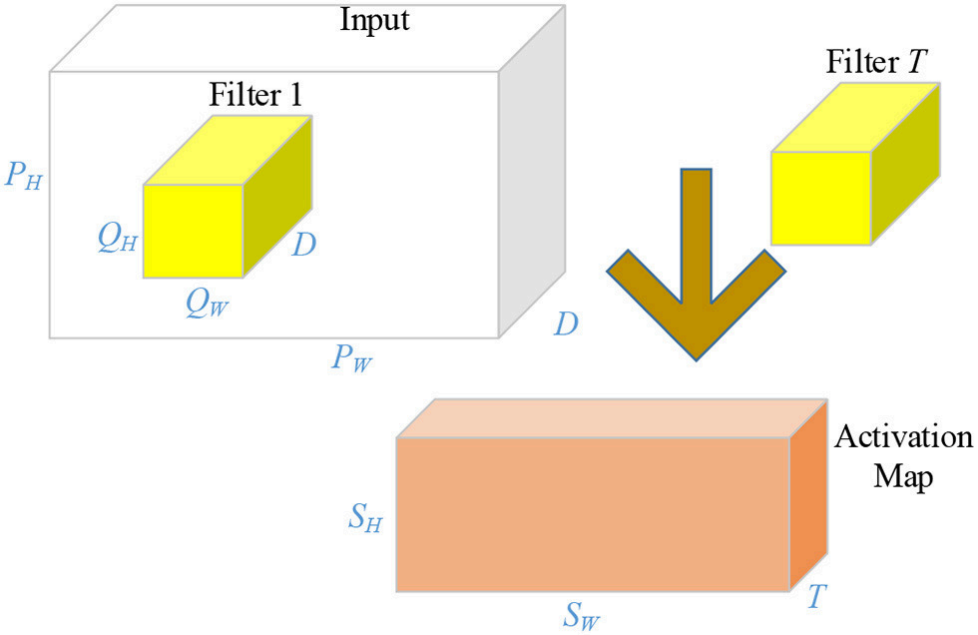
Illustration of convolution operation.

Compared to traditional neural networks, there are several advanced techniques used in AlexNet. First, CLs contain a set of learnable filters. For example, the user has a 3D input with a size of *P*_*W*_ × *P*_*H*_ × *D*, a 3D filter with a size of *Q*_*W*_ × *Q*_*H*_ × *D*. As a consequence, the size of the output activation map is *S*_*W*_ × *S*_*H*_. The value of *S*_*W*_ and *S*_*H*_ can be obtained by

(1) $$\begin{array}{l} S_{W}=1+\frac{P_{W}-Q_{W}+2\beta }{\mu } \end{array}$$

(2) $$\begin{array}{l} S_{H}=1+\frac{P_{H}-Q_{H}+2\beta }{\mu } \end{array}$$

where μ is the stride size and β represents the margin. Commonly, there may be *T* filters. One filter will generate one 2D feature map, and *T* filters will yield an activation map with a size of *S*_*W*_ × *S*_*H*_ × *T*. An illustration of convolutional procedure is shown in Figure 3. The “feature learning” in the filters here, can be regarded as a replacement of the “feature extraction” in traditional machine learning.

Second, the rectified linear unit (ReLU) function was employed to replace the traditional sigmoid function *S*(*x*) in terms of the activation function (31). The reason is because the sigmoid function may come across a gradient vanishing problem in deep neural network models.

(3) $$\begin{array}{l} S\left(x\right)=\frac{1}{1+\exp\left(-x\right)} \end{array}$$

Therefore, the ReLU was proposed and defined as follows:

(4) ReLU(x)=max(0,x)

The gradient of ReLU is one at all times, when the input is larger than or equal to zero. Scholars have proven that the convergence speed of deep neural networks, with ReLU as the activation function, is 6x quicker than traditional activation functions. Therefore, the new ReLU function greatly accelerates the training procedure.

**Figure 4:**
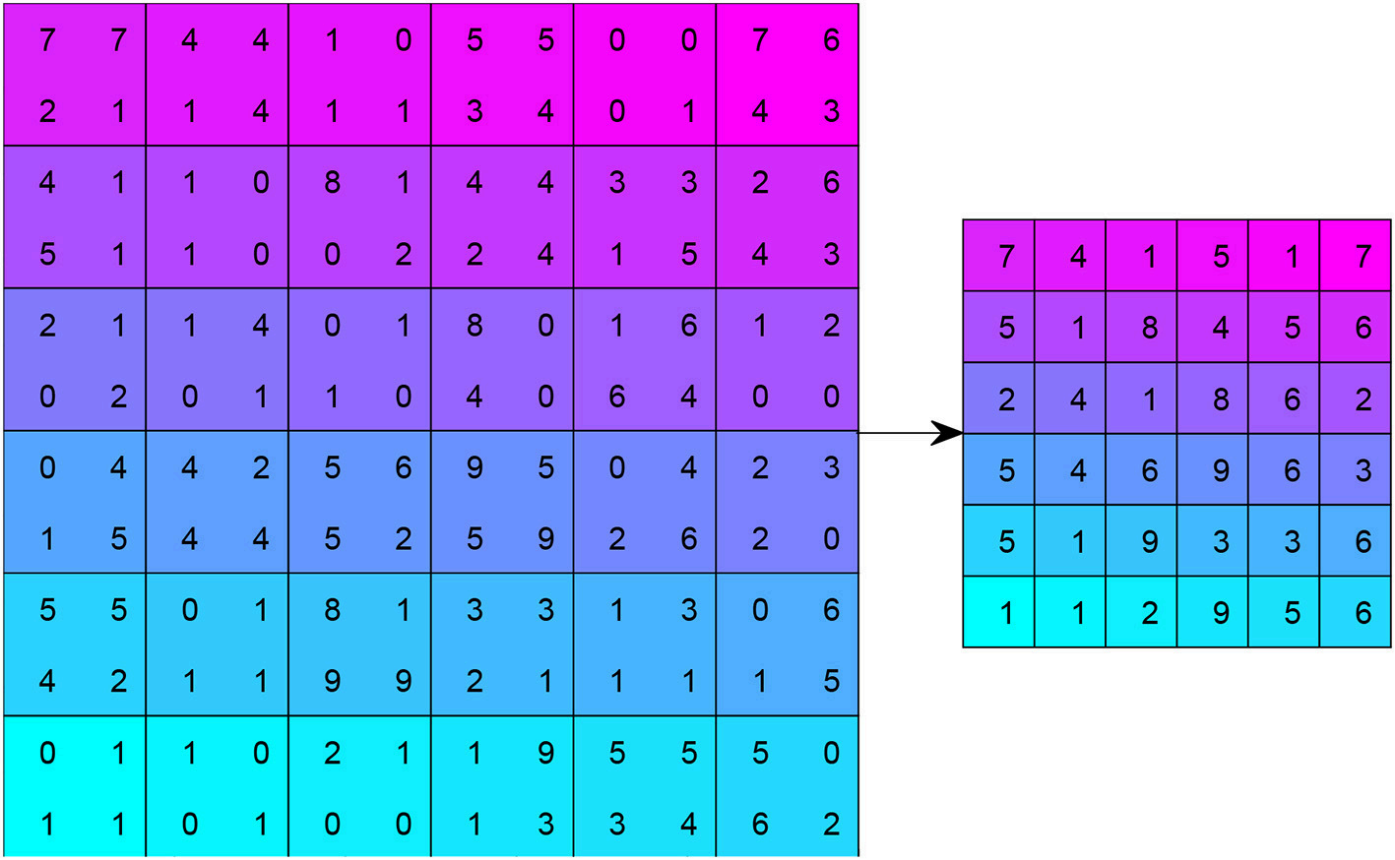
Example of max pooling (stride = 2, kernels size = 2).

Third, a pooling operation is implemented with two advantages: (i) It can reduce the size of the feature map, and thus reduce the computation burden. (ii) It ensures that the representation becomes invariant to the small translation of the input. Map pooling (MP) is a common technique that chooses the maximum value among a 2 × 2 region of interest. Figure 4 shows a toy example of MP, with a stride of 2 and kernel size of 2.

The fourth improvement is the “local response normalization (LRN).” Krizhevsky et al. (26) proposed the LRNs in order to aid generalization. Suppose that *a*_*i*_ represents a neuron computed by applying kernel *i* and ReLU non-linearity, then the response-normalized neuron *b*_*i*_ will be expressed as:

(5) $$\begin{array}{l} b_{i}=\frac{a_{i}}{{\left(m+\alpha \overset{\min\left(Z-1,i+z/2\right)}{\underset{s=\max\left(0,i-z/2\right)}{\sum }}a_{s}^{2}\right)}^{\beta }} \end{array}$$

where *z* is the window channel size, controlling the number of channels used for normalization of each element, and *Z* is the gross number of kernels in that layer. Hyperparameters are set as: β = 0.75, α = 10^−4^, *m* = 1, and *z* = 5.

**Figure 5:**
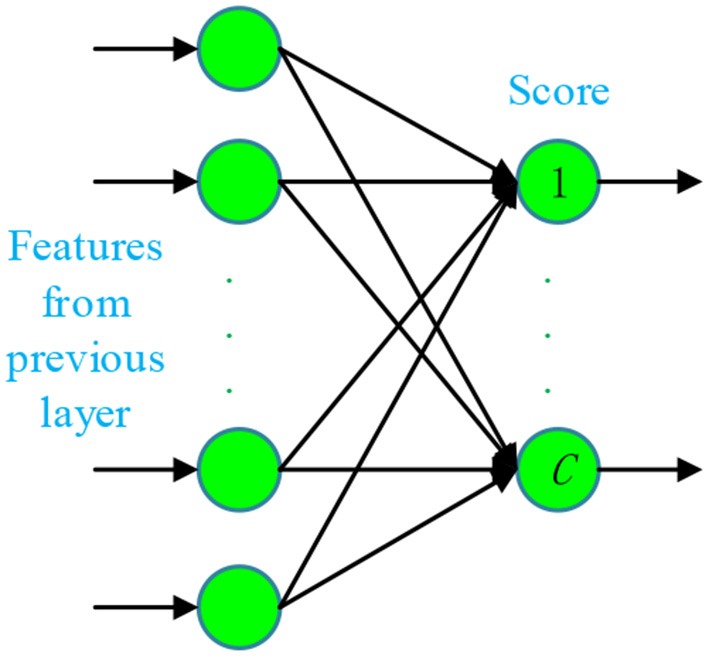
Structure of last fully-connected layer (*C* stands for the number of total classes).

Fifth, the fully connected layers (FCLs) have connections to all activations in the previous layer, so they can be modeled as multiplying the input by a weight matrix and then adds a bias vector. The last fully-connected layer includes the equal number of artificial neurons as the number of total classes *C*. Therefore, each neuron in the last FCL represents the score of that cognate class, as shown in Figure 5.

**Figure 6:**
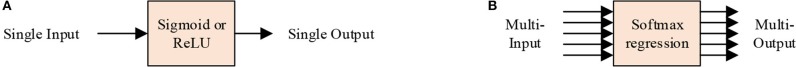
Two modes of activation function. **(A)** Single input single output mode. **(B)** Multiple input multiple output mode.

Sixth, the softmax layer (SL), utilizes the multiclass generalization of logistic regression (32), also known as softmax function. SL is commonly connected after the final FCL. From the perspective of the activation function, the sigmoid/ReLU function works on a single input single output mode, while the SL serves as a multiple input multiple output mode, as shown in Figure 6. A toy example can be imagined. Suppose we have a four input at the final SL layer with values of (1–4), then after a softmax layer, we have an output of [0.032, 0.087, 0.236, 0.643].

Suppose that *T*(*f*) symbolizes the prior class probability of class *f*, and *T*(*h*|*f*) means the conditional probability of sample *h* given class *f*. Then we can conclude that the likelihood of sample *h* belonging to class *f* is

(6) $$\begin{array}{l} T\left(f|h\right)=\frac{T\left(h|f\right)\times T\left(f\right)}{\overset{F}{\underset{i=1}{\sum }}T\left(h|i\right)\times T\left(i\right)} \end{array}$$

Here *F* stands for the whole number of classes. Let Ω_*f*_ equals

(7) $$\begin{array}{l} \Omega_{f}=\ln\;\left[T\left(h,f\right)\times T\left(f\right)\right] \end{array}$$

Afterwards, we get

(8) $$\begin{array}{l} T\left(f|h\right)=\frac{\exp\left(\Omega_{f}\left(h\right)\right)}{\overset{F}{\underset{i=1}{\sum }}\exp\left(\Omega_{i}\left(h\right)\right)} \end{array}$$

Finally, a dropout technique is used, since training a big neural network is too expensive. Dropout freezes neurons at random with a dropout probability (*P*_*D*_) of 0.5. During training phase, those dropped out neurons are not engaged in both a forward and backward pass. During the test phase, all neurons are used but with outputs multiplied by *P*_*D*_ of 0.5 (33).

**Figure 7:**
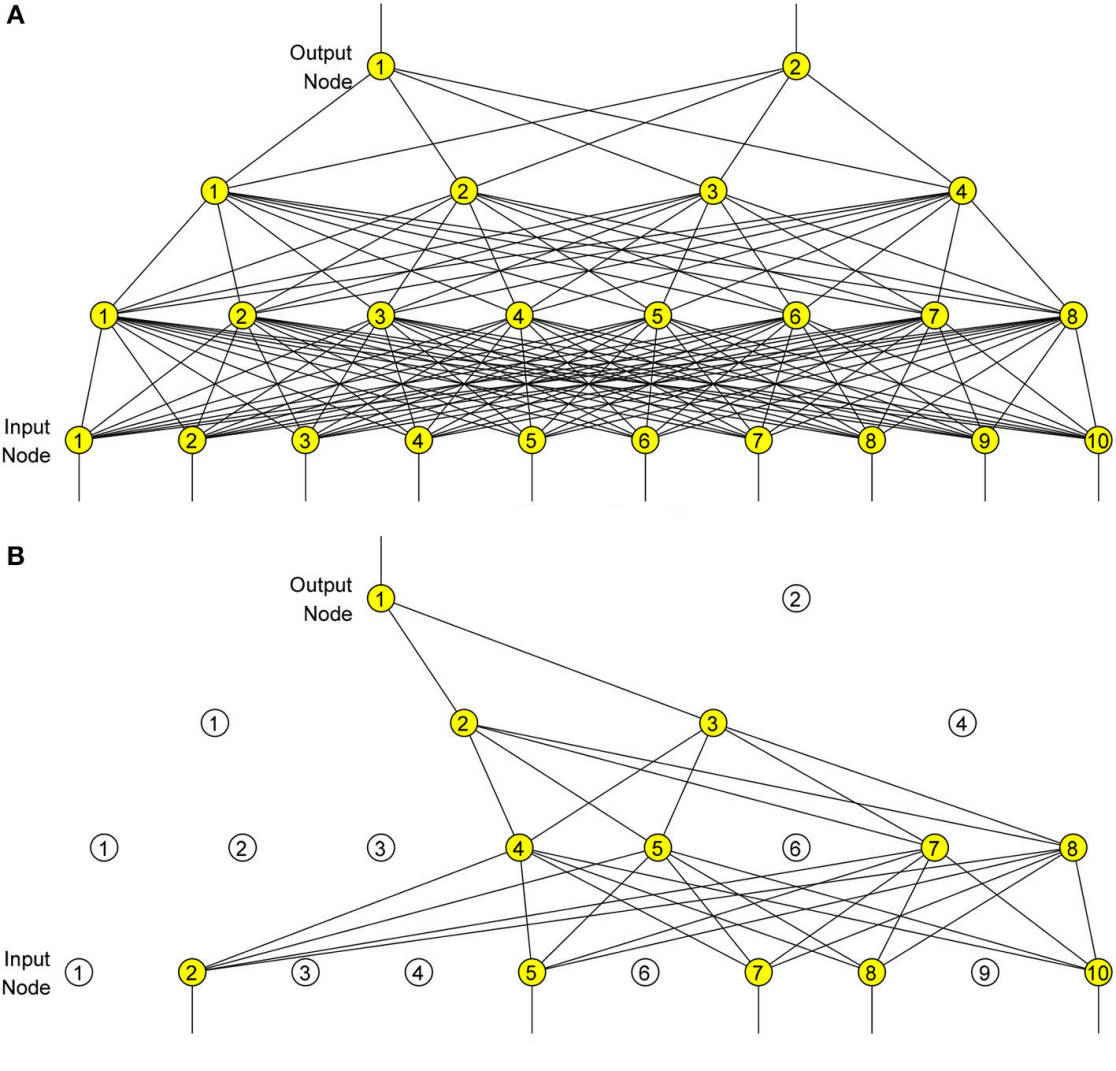
Dropout neural network. **(A)** Before dropout. **(B)** After dropout.

It can be regarded as taking a geometric mean of predictive distributions, generated by exponentially-many small-size dropout neural networks. Figure 7A shows a plain neural network with numbers of neurons at each layer as (2, 4, 8, 10), and Figure 7B shows the corresponding dropout neural network with *P*_*D*_ of 0.5, where only (1, 2, 4, 5) neurons remain active at each layer.

### Transfer AlexNet to Alcoholism Identification

First, we needed to modify the structure. The last FCL was revised, since the original FCLs were developed to classify 1,000 categories. Twenty randomly selected classes were listed as: scale, barber chair, lorikeet, miniature poodle, Maltese dog, tabby, beer bottle, desktop computer, bow tie, trombone, crash helmet, cucumber, mailbox, pomegranate, Appenzeller, muzzle, snow leopard, mountain bike, padlock, diamondback. We observed that none of them are related to the brain image. Hence, we could not directly apply AlexNet as the feature extractor. Therefore, fine-tuning was necessary.

Since the length of output neurons in orthodox AlexNet (1000) is not equal to the number of classes in our task (2), we needed to revise the corresponding softmax layer and classification layer. The revision is shown in Table 4. In our transfer learning scheme, we used a new randomly-initialized fully connected layer with two neurons, a softmax layer, and a new classification layer with only two classes (alcoholism and non-alcoholism).

**Table 4:** Revision of Last three layers of AlexNet.

**Layer**	**Original**	**Replaced**
23	FCL (1000) with pre-trained weights and biases	FCL (2) with random initialization
24	Softmax Layer	Softmax Layer
25	Classification Layer (1,000 classes)	Classification Layer (two classes: alcoholism and non-alcoholism)

Next, we set the training options. Three subtleties were checked before training. First, the whole training epoch should be small for a transfer learning. In this study, we set the number of training epochs to 10. Second, the global learning rate was set to a small value of 10^−4^ to slow learning down, since the early parts of this neural network were pre-trained. Third, the learning rate of new layers were 10 times that of the transferred layer, since the transferred layers with pre-trained weights/biases and new layers were with random-initialized weights/biases.

**Figure 8:**
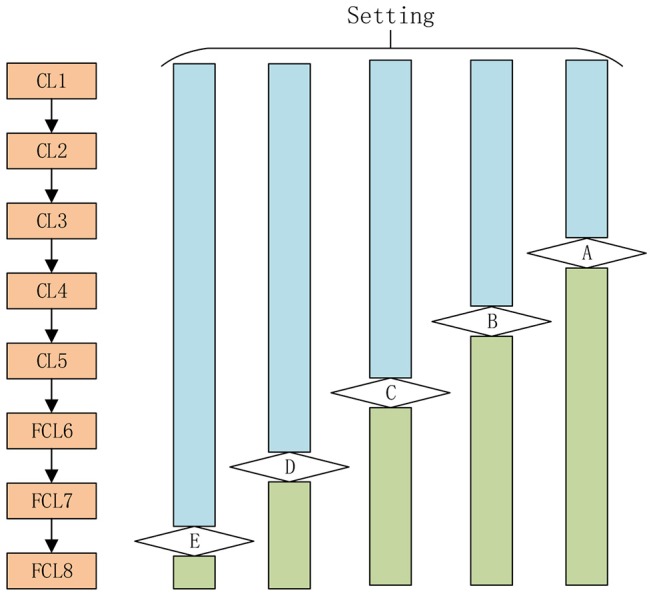
Five different settings A-E (Setting A stands for the layers from first layer till layer A are transferred layers, and the remaining layers are replaced layers).

Third, we varied the numbers of transferred layers and tested different settings. The AlexNet consists of five conv layers (CL1, CL2, CL3, CL4, and CL5) and three fully-connected layers (FCL6, FL7, FL8). As a result, we tested five different transfer learning settings as shown in Figure 8 in total, in all experiments. For example, here Setting A means that the layers from the first layer to layer A are transferred directly with learning rate as 10^−4^ × 1 = 10^−4^. The late layers from layer A to the last layer are randomly initialized with a learning rate of 10^−4^ × 10 = 10^−3^.

### Implementation and Measure

We ran the experiment many times. Each time, the training-validation-test division was set at random again. The training procedure stopped when either the algorithm reached maximum epoch, or the performance of validation decreased over a preset training epoch. We repeatedly tuned the hyperparameters and found those optimal hyper-parameters based on a validation set. After the hyperparameters were fixed, we ran the final model on the test set for 10 runs. The test set confusion matrix across all runs was recorded, and the following five measures were calculated: sensitivity (SEN), specificity (SPC), precision (PRC), accuracy (ACC), and F1 score. Assume TP, TN, FP, and FN stands for true positive, true negative, false positive, and false negative, respectively, all five measures were defined as:

(9) $$\begin{array}{l} SEN=\frac{TP}{TP+FN} \end{array}$$

(10) $$\begin{array}{l} SPC=\frac{TN}{TN+FP} \end{array}$$

(11) $$\begin{array}{l} PRC=\frac{TP}{TP+FP} \end{array}$$

(12) $$\begin{array}{l} ACC=\frac{TP+TN}{TP+TN+FP+FN} \end{array}$$

F_1_ considers both the precision and the sensitivity to computer the score (34). That means, the measure of the “F1 score” is the harmonic mean of the previous two measures: precision and sensitivity.

(13) $$\begin{array}{l} F_{1}={\left(\frac{SEN^{-1}+PRC^{-1}}{2}\right)}^{-1} \end{array}$$

Using simple mathematical knowledge, we can obtain:

(14) $$\begin{array}{l} \begin{array}{c} F_{1}=2/\left(\frac{TP+FN}{TP}+\frac{TP+FP}{TP}\right)\\ =2/\left(\frac{2TP+FP+FN}{TP}\right)\\ =\frac{2\times TP}{2\times TP+FP+FN} \end{array} \end{array}$$

Then, the average and standard deviation (SD) of all five measures of 10 runs of the test set were calculated and used for comparison. For ease of understanding, a pseudocode of our experiment is listed below in Table 5. The first block is to split the dataset into non-test and test sets. In the second block, the non-test set was split into training and validation randomly. The performance of the retrained AlexNet model was recorded and used to select the optimal transfer learning setting S^*^. In the final block, the performance on the test set via the retrained AlexNet using setting S^*^ was recorded and outputted.

**Table 5:** Pseudocode of our experiment.

[NonTest, Test] = split(Dataset);
for S = [A, B, C, D, E]
for i = 1:10
[train(i), valid(i)] = split(NonTest),
Model(S, i) = TrainNetwork(AlexNet, train(i), valid(i), Setting = S),
PerfValid(S, i) = Predict(Model(S, i), valid(i)),
end
PerfValid(S) = mean(PerfValid(S, i)),
End
S* = argmax[Performance(S)],
for i = 1:10
[train(i), valid(i)] = split(NonTest),
Model(S*, i) = TrainNetwork(AlexNet, train(i), valid(i), Setting = S*),
PerfTest(S*, i) = predict(Model(S*, i), Test),
End
PerfTest(S*) = mean(PerfTest(S*, i)),
Output PerfTest(S*),

## Results

### Data Augmentation Results

**Figure 9:**
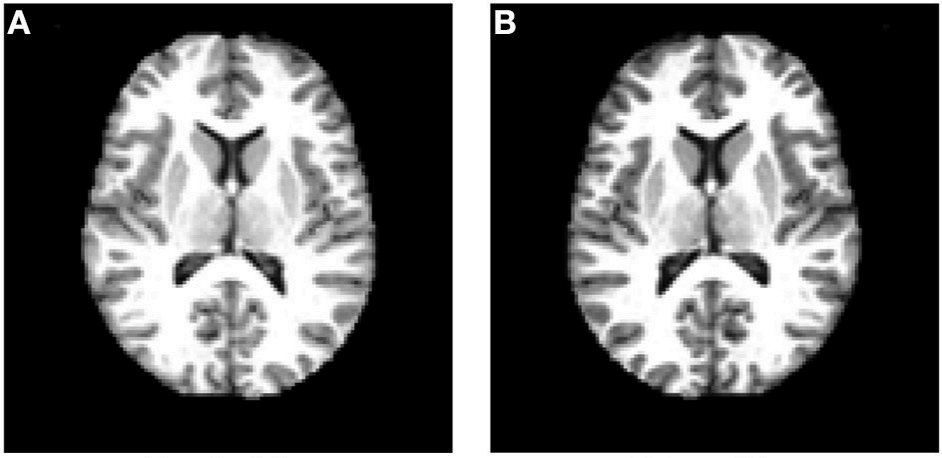
Data augmentation by horizontal flipping. **(A)** Original image. **(B)** Flipped image.

**Figure 10:**
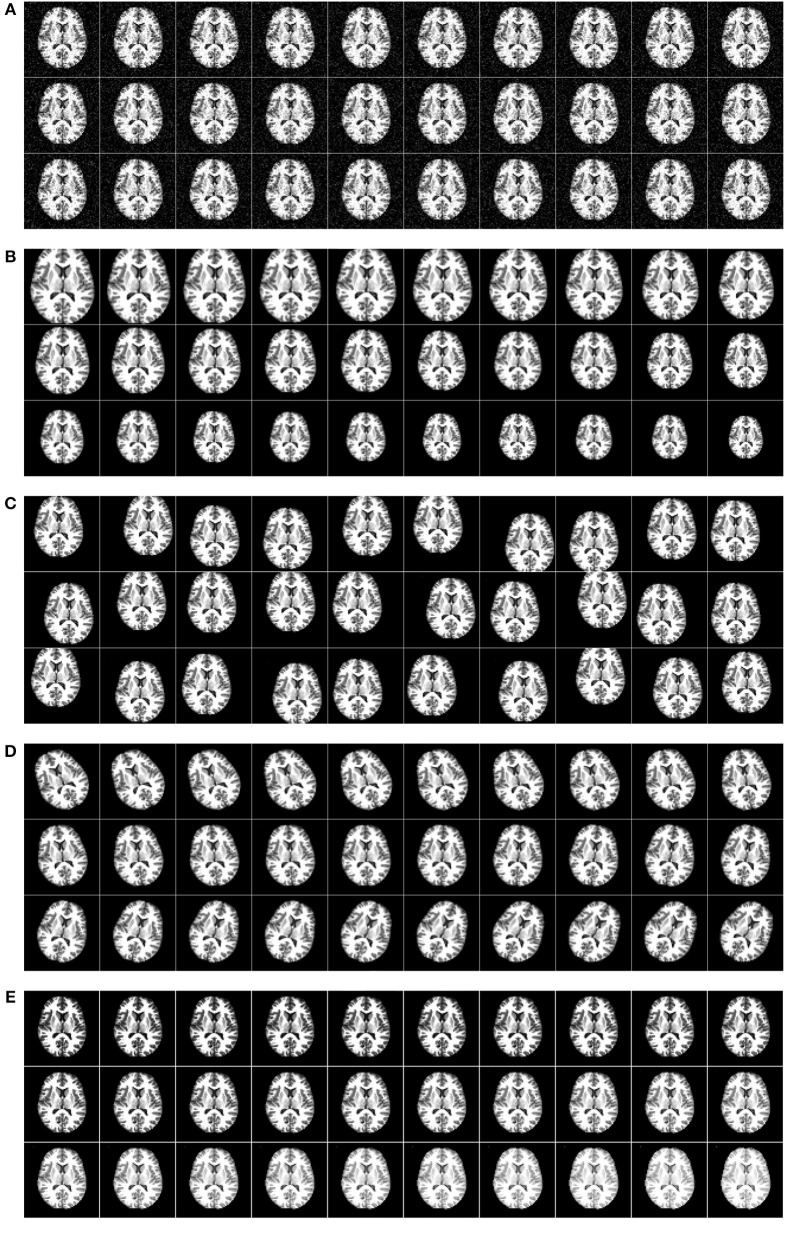
Five augmentation techniques of the original image. **(A)** Noise injection. **(B)** Scaling. **(C)** Random translation. **(D)** Image rotation. **(E)** Gamma correction.

Figure 9 shows the horizontally flipped image. Here, vertical flipping was not carried out because it can be seen as a combination of horizontal flipping with 180-degree rotation. Figure 10 shows the data augmentation results of five different techniques: (a) noise injection; (b) scaling; (c) random translation; (d) image rotation; (e) Gamma correction. Due to the page limit, the data augmentation results on the flipped image are not shown.

### Comparison of Setting of TL

In this experiment, we compared five different TL settings on the validation set. The results of Setting A are shown in Table 6, where the last row shows the mean and standard deviation value. The results of Setting E are shown in Table 7. Due to page limit, we only show the final results of Setting B, C, and D in Table 8.

**Table 6:** Ten runs of validation performance of transfer learning using Setting A.

**Run**	**SEN**	**SPC**	**PRC**	**ACC**	**F1**
1	96.67	93.33	93.54	95.00	95.05
2	100.00	100.00	100.00	100.00	100.00
3	90.00	100.00	100.00	95.00	94.70
4	96.67	90.00	90.63	93.33	93.55
5	90.00	96.67	96.43	93.33	93.10
6	96.67	96.67	96.67	96.67	96.67
7	96.67	96.67	96.88	96.67	96.66
8	96.67	100.00	100.00	98.33	98.28
9	100.00	90.00	90.99	95.00	95.26
10	96.67	93.33	93.54	95.00	95.05
Mean ± *SD*	96.00 ± 3.27	95.67 ± 3.67	95.87 ± 3.40	95.83 ± 2.01	95.83 ± 2.00

**Table 7:** Ten runs of validation performance of transfer learning using Setting E.

**Run**	**SEN**	**SPC**	**PRC**	**ACC**	**F1**
1	93.33	100.00	100.00	96.67	96.55
2	100.00	96.67	96.88	98.33	98.39
3	100.00	100.00	100.00	100.00	100.00
4	100.00	100.00	100.00	100.00	100.00
5	93.33	93.33	93.33	93.33	93.33
6	96.67	100.00	100.00	98.33	98.28
7	100.00	100.00	100.00	100.00	100.00
8	96.67	93.33	93.54	95.00	95.05
9	96.67	93.33	93.54	95.00	95.05
10	100.00	100.00	100.00	100.00	100.00
Mean ± *SD*	97.67 ± 2.60	97.67 ± 3.00	97.73 ± 2.93	97.67 ± 2.38	97.67 ± 2.37

**Table 8:** Comparison of different setting.

**Setting**	**SEN**	**SPC**	**PRC**	**ACC**	**F1**
A	96.00 ± 3.27	95.67 ± 3.67	95.87 ± 3.40	95.83 ± 2.01	95.83 ± 2.00
B	96.33 ± 3.79	96.00 ± 2.49	96.12 ± 2.43	96.17 ± 2.36	96.15 ± 2.43
C	96.33 ± 3.48	96.33 ± 3.14	96.49 ± 2.94	96.33 ± 2.08	96.33 ± 2.11
D	97.00 ± 3.79	97.00 ± 2.77	97.06 ± 2.70	97.00 ± 2.56	96.98 ± 2.62
**E**	**97.67 ± 2.60**	**97.67 ± 3.00**	**97.73 ± 2.93**	**97.67 ± 2.38**	**97.67 ± 2.37**

*Bold means the best*.

**Figure 11:**
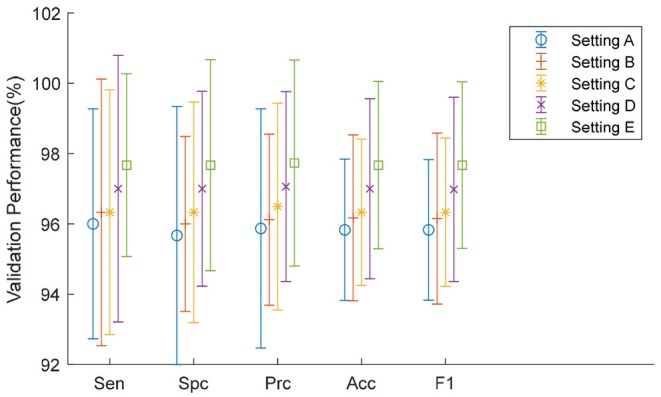
Error bar of five TL settings.

Here, it can be seen from Table 8 that Setting E, i.e., replacing the FCL8, achieves the greatest performance among all five settings with respect to all measures. The reason may be (i) we expanded a relatively small dataset to a large training set using data augmentation; and (ii) the dissimilarity of our data and the original 1,000-category dataset. The first fact ensures that retraining avoids overfitting; and the latter fact suggests that it is more practical to put most of the layers initialized with weights from a pretrained model, than freezing those layers. For clarity, we plotted the error bar and show it in Figure 11.

### Analysis of Optimized TL Setting

The structure of the optimal transfer learning model (Setting E) is listed in Table 9. Compared to the traditional AlexNet model, the weights and biases of FCL8 were reduced from 4,096,000 to 8,192, and from 1,000 to 2, respectively. The main reason is that we only had two categories in our classification task. Thus, the whole weight of the deep neural network reduced slightly from 60,954,656 to 56,866,848.

**Table 9:** Learnable layers in optimal transfer learning model.

**Name**	**Weights**	**Weights (%)**	**Biases**	**Biases (%)**
CL1 (Ours)	11*11*3*96 = 34,848	0.06	1*1*96 = 96	1.00
CL2 (Ours)	5*5*48*256 = 307,200	0.54	1*1*256 = 256	2.68
CL3 (Ours)	3*3*256*384 = 884,736	1.56	1*1*384 = 384	4.01
CL4 (Ours)	3*3*192*384 = 663,552	1.17	1*1*384 = 384	4.01
CL5 (Ours)	3*3*192*256 = 442,368	0.78	1*1*256 = 256	2.68
FCL6 (Ours)	4096*9216 = 37,748,736	66.38	4096*1 = 4,096	42.80
FCL7 (Ours)	4096*4096 = 16,777,216	29.50	4096*1 = 4,096	42.80
FCL8 (AlexNet)	1000*4096 = 4,096,000		1000*1 = 1,000	
FCL8 (Ours)	2*4096 = 8,192	0.01	2*1 = 2	0.02
CL Subtotal (AlexNet)	2,332,704		1,376	
CL Subtotal (Ours)	2,332,704	4.10	1,376	14.38
FCL Subtotal (AlexNet)	58,621,952		9,192	
FCL Subtotal (Ours)	54,534,144	95.90	8,194	85.62
Total (AlexNet)	60,954,656		10,568	
Total (Ours)	56,866,848	100	9,570	100

Nevertheless, we can observe that FCL6 and FCL7 still constitutes too many weights and biases. For example, FCL6 occupied 37,748,736/56,866,848 = 66.38% of the total weights in this optimal model, and FCL7 occupied 16,777,216/56,866,848 = 29.50% of the total weights. Additionally, the FCL subtotal comprised 95.90% of the total weights. This is the main limitation of our method. To solve it, we need to replace the fully connected layers with 1 × 1 conv layers. Another solution is to choose small-size transfer learning models, such as SqueezeNet, ResNet, GoogleNet, etc.

### Effect of Data Augmentation

This experiment compared the performance of runs with data augmentation against runs without data augmentation (DA). Configuration of transfer learning was set to Setting E. All the other parameters and network structures were the same as the previous experiments. The performance of the 10 runs without using DA are shown in Table 10. The results in terms of all measures are equal to or slightly above 95%.

**Table 10:** Ten runs without using data augmentation (Setting E).

**Run**	**SEN**	**SPC**	**PRC**	**ACC**	**F1**
1	83.33	96.67	96.15	90.00	89.29
2	96.67	93.33	93.54	95.00	95.05
3	96.67	93.33	93.54	95.00	95.05
4	96.67	90.00	90.78	93.33	93.54
5	96.67	100.00	100.00	98.33	98.28
6	96.67	96.67	96.67	96.67	96.67
7	96.67	93.33	93.54	95.00	95.05
8	93.33	100.00	100.00	96.67	96.55
9	93.33	96.67	96.67	95.00	94.94
10	100.00	93.33	93.75	96.67	96.77
Mean ± *SD*	95.00 ± 4.28	95.33 ± 3.06	95.46 ± 2.84	95.17 ± 2.17	95.12 ± 2.32

The comparison of using DA against not using DA is shown in Table 11. We can discern that DA indeed enhances the classification performance. The reason is that having a large dataset is crucial for good performance. The alcoholism image dataset is commonly of small size, and its size can be augmented to the order of tens of thousands (48,320 in this study). AlexNet can make full use of all its parameters with a big dataset. Without using DA, overfitting is likely to occur in the transferred model.

**Table 11:** Effect of using data augmentation technique.

**DA**	**SEN**	**SPC**	**PRC**	**ACC**	**F1**
Not use DA	95.00 ± 4.28	95.33 ± 3.06	95.46 ± 2.84	95.17 ± 2.17	95.12 ± 2.32
Use DA (ours)	97.67 ± 2.60	97.67 ± 3.00	97.73 ± 2.93	97.67 ± 2.38	97.67 ± 2.37

### Results of Proposed Method

In this experiment, we chose Setting E (replace the final block) as shown in Figure 8. Here, the retrained neural network was tested on the test set. The results over all 10 runs on the test set are listed in Table 12 with details of sensitivity, specificity, precision, accuracy, and the F1 score of each run. Setting E yielded a sensitivity of 97.44 ± 1.15%, a specificity of 97.41 ± 1.51%, a precision of 97.34 ± 1.49%, an accuracy of 97.42 ± 0.95%, and an F1 score of 97.37% ± 0.97%. Comparing Table 12 with Table 7, we can see that the mean value of test performance is slightly worse than that of the validation performance, but the standard deviation of the test performance is much smaller than that of the validation performance.

**Table 12:** Ten runs of proposed method on the test set (Setting E).

**Run**	**SEN**	**SPC**	**PRC**	**ACC**	**F1**
1	97.44	96.31	96.22	96.86	96.82
2	98.72	93.81	93.93	96.23	96.25
3	94.87	96.31	96.09	95.61	95.47
4	97.44	98.75	98.72	98.11	98.07
5	98.72	98.75	98.72	98.73	98.72
6	98.72	97.53	97.47	98.11	98.09
7	97.44	98.78	98.72	98.12	98.07
8	97.44	98.75	98.75	98.12	98.05
9	96.15	97.53	97.40	96.84	96.74
10	97.44	97.53	97.44	97.48	97.44
Mean ± *SD*	97.44 ± 1.15	97.41 ± 1.51	97.34 ± 1.49	97.42 ± 0.95	97.37 ± 0.97

### Comparison to Alcoholism Identification Approaches

**Figure 12:**
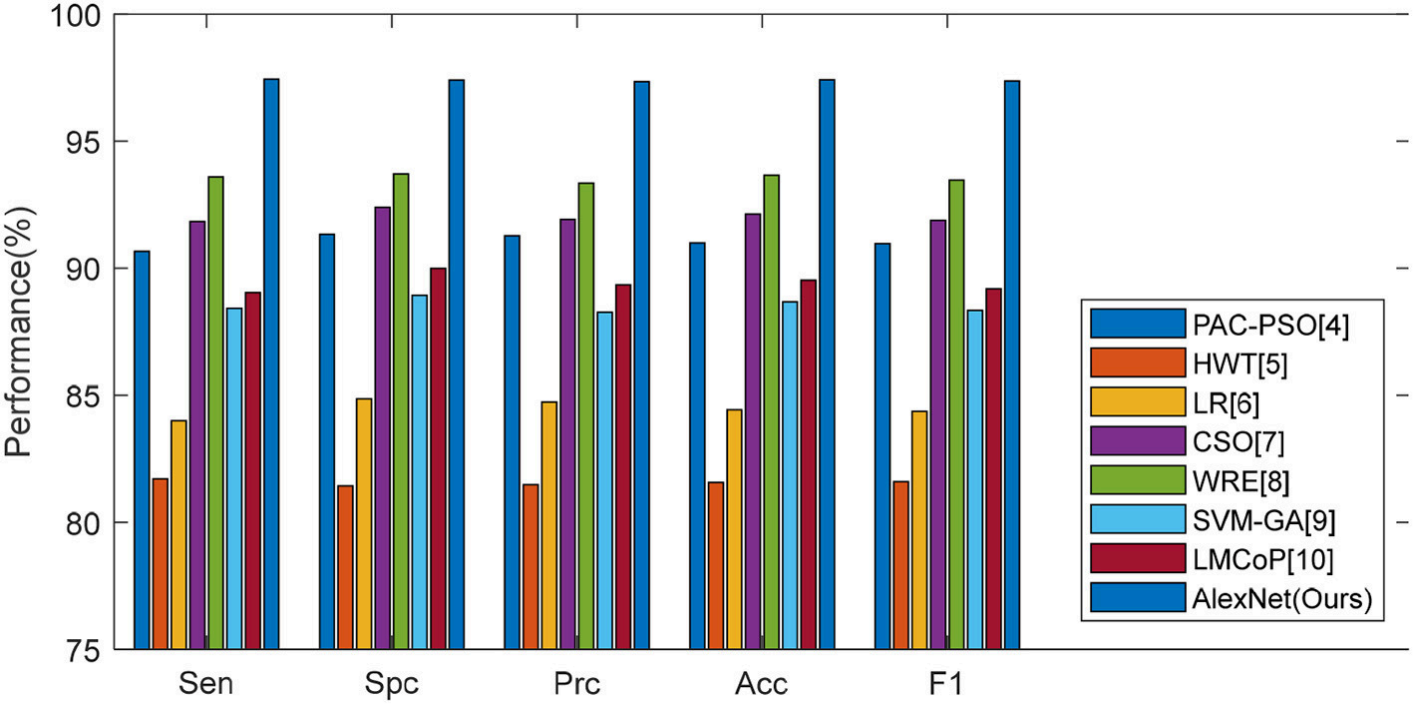
Bar plot of comparison of eight algorithms.

This proposed transfer learning approach was compared with seven state-of-the-art approaches: PAC-PSO (4), HWT (5), LR (6), CSO (7), WRE (8), SVM-GA (9), and LMCoP (10). The comparison results are shown in Table 13. The cognate bar plot is shown in Figure 12. We can observe that our AlexNet transfer learning model has more than 3% improvement compared to the next best approach.

**Table 13:** Comparison with state-of-the-art approaches.

**Approach**	**SEN**	**SPC**	**PRC**	**ACC**	**F1**
PAC-PSO (4)	90.67	91.33	91.28	91.00	90.97
HWT (5)	81.71	81.43	81.48	81.57	81.60
LR (6)	84.00	84.86	84.73	84.43	84.36
CSO (7)	91.84	92.40	91.92	92.13	91.88
WRE (8)	93.60	93.72	93.35	93.66	93.47
SVM-GA (9)	88.42	88.93	88.27	88.68	88.34
LMCoP (10)	89.04	90.00	89.35	89.53	89.19
AlexNet (Ours)	97.44	97.41	97.34	97.42	97.37

The reason is that this proposed model did not need to find features manually; nevertheless, it only used a learned feature from a pretrained model as initialization, and utilized the enhanced training set to fine-tune those learned features. This has two advantages: First, the development is quite fast, which can be reduced to <1 day. Second, the features can be fine-tuned to be more appropriate to this alcoholism classification task than other manually-designated features.

The bioinspired-algorithm may help retraining our AlexNet model. Particle swarm optimization (PSO) (35–37) and other methods will be tested. Cloud computing (38) in particular can be integrated into our method, to help diagnosis of remote patients.

## Conclusions

In this study, we proposed an AlexNet-based transfer learning method and applied it to the alcoholism identification task. This paper may be the first paper using transfer learning in the field of alcoholism identification. The results showed that this proposed approach achieved promising results with a sensitivity of 97.44 ± 1.15%, a specificity of 97.41 ± 1.51%, a precision of 97.34 ± 1.49%, an accuracy of 97.42 ± 0.95%, and an F1 score of 97.37 ± 0.97.

Future studies may include the following points: (i) other deeper transfer learning models, such as ResNet, DenseNet, GoogleNet, SqueezeNet, etc. should be tested; (ii) other data augmentation techniques should be attempted. Currently our dataset is small, so data augmentation may have a distinct effect on improving the performance; (iii) how to set the learning rate factor of each individual layer in the whole neural network, remains a challenge and needs to be solved; (iv) this method is ready to run on a larger dataset and can assist radiologists in their routine screening of brain MR images.

## Data Availability

The datasets for this manuscript are not publicly available because we need approval from our affiliations. Requests to access the datasets should be directed to yudongzhang@ieee.org.

## Author Contributions

S-HW and Y-DZ conceived the study. SX and XC designed the model. CT, JS, and DG analyzed the data. S-HW, XC, and Y-DZ acquired the preprocessed data. SX and CT wrote the draft. S-HW, CT, JS, and Y-DZ interpreted the results. DG provided English revision of this paper. All authors provided critical revision and consent for this submission.

### Conflict of Interest Statement

The authors declare that the research was conducted in the absence of any commercial or financial relationships that could be construed as a potential conflict of interest.
